# Limited evidence of hierarchical encoding in the cheerleader effect

**DOI:** 10.1038/s41598-019-45789-6

**Published:** 2019-06-27

**Authors:** Daniel J. Carragher, Nicole A. Thomas, O. Scott Gwinn, Mike E. R. Nicholls

**Affiliations:** 1 College of Education, Psychology, and Social Work, Flinders University, Adelaide, Australia; 2 School of Psychological Sciences, Monash University, Melbourne, Australia

**Keywords:** Human behaviour, Social behaviour

## Abstract

“The cheerleader effect” refers to the increase in attractiveness that an individual face experiences when seen in a group of other faces. It has been proposed that the cheerleader effect occurs because (a) the visual system rapidly summarises a group of faces into an ensemble representation, (b) which is hypothesised to be highly attractive because of its average facial characteristics, and (c) observers remember individual faces to be more alike the ensemble representation than they were, due to hierarchical structure of visual working memory. Across three experiments, we investigated whether the cheerleader effect is consistent with hierarchical encoding, by asking observers to give attractiveness ratings to the same target faces shown in groups and alone. Consistent with hierarchical encoding, the largest attractiveness increases of 1.5–2.0% occurred when target faces were presented in groups of faces that could be mentally summarised to create an ensemble representation with average facial characteristics. Surprisingly, smaller cheerleader effects still occurred in conditions that were incompatible with hierarchical encoding (i.e., groups with non-human images). Together, these results offer only limited evidence for the role of hierarchical encoding in the cheerleader effect, suggesting that alternative mechanisms must be explored in future research.

## Introduction

Facial attractiveness is rapidly evaluated during first impressions^1–3^. Traditionally, attractiveness is associated with physiological traits in the face, including symmetry^4–6^, sexually dimorphic appearance^7–9^, and averageness^10–12^. Yet, a growing number of recent studies have found that the perceived attractiveness of an individual face can fluctuate in response to cues that are external to the face itself, such as social context^13–19^. One prominent example of the effect that social context has on judgments of facial attractiveness is known colloquially as “the cheerleader effect”^20^. The cheerleader effect is said to occur when the same face is perceived to be more attractive when seen in a group, compared to when it is seen alone^13,16,19^. Walker and Vul^16^ provided the first empirical support for the cheerleader effect, showing that the phenomenon occurs for both female and male faces, seen in groups of 3 to 16 faces. Carragher, *et al*.^13^ were the first to replicate the cheerleader effect, finding that target faces were consistently perceived to be approximately 1.5–2.0% more attractive when presented in a group compared to alone, regardless of the spatial position of the target face within the group. Walker and Vul^16^ hypothesised that the cheerleader effect was the result of a complex interaction between the way that visual scenes are summarised by the visual system^21,22^, the attractiveness of average faces^8,12^, and the hierarchical structure of visual working memory^23,24^.

When presented with a complex visual scene, the human visual system rapidly creates a summary representation of the display, through a process known as ensemble coding^21,22,25,26^. Ensemble coding allows the visual system to minimise the cognitive load associated with encoding many individual items from a display, whilst allowing the observer to retain access to the accurate summary statistics of the scene^21,22,26^. Through ensemble coding, observers can accurately identify the average characteristics of a scene, such as the average orientation of a set of tilted gabor patches^27^ or the average size of a set of circles^25,28,29^. Ensemble coding occurs rapidly^25,29,30^, and summary statistics are extracted with remarkable accuracy^21,22^, even from unattended^31,32^ or crowded displays^27,33^. Importantly, ensemble coding also occurs for groups of faces^34–36^.

Through ensemble coding, observers can accurately extract the average identity of a group of faces^37–39^. Neumann, *et al*.^38^ presented observers with groups of faces consisting of four different identities. The observers were then asked whether the subsequent memory probe showed a face that was a member of the preceding group. In addition to correctly recognising the individual faces that had been members of the group, observers also mistakenly “recognised” the set average (i.e., a face that had been created by digitally averaging the individual faces in the group together). Crucially, observers did not falsely recognise “foil averages” (i.e., a face made by averaging a different group of faces), suggesting that the set average is not mistakenly recognised because average faces are inherently familiar^40^, but because the observer mentally summarises the group and extracts its average identity^37,38^.

Ensemble coding also facilitates the accurate identification of the average emotional expression shown by a group of faces^34,36,41,42^. Remarkably, even though no individual face shows the average emotional expression of the group, observers can identify the group’s average expression with the same precision that is shown when identifying the expression of a single emotional face^41^. The ensemble coding of expression occurs rapidly, as observers can accurately identify the average expression shown by a group of 16 faces that has been presented for just 50 ms^35,36,42^. In addition to rapidly extracting the average emotion^35,36,41^ and identity^37–39^ from groups of faces, observers can accurately summarise other characteristics of faces, including average ethnicity and dominance^43^, gender^36^, and attractiveness^44^. Although ensemble coding allows observers to rapidly extract the ‘gist’ of complex visual scenes^21,22,26^, this process influences the accuracy with which the characteristics of individual items from the group are remembered^23,24,45^.

When asked to recall the characteristics of a single item from a group, observers remember it to be more alike the ensemble representation than it truly was^23,45,46^. For example, Brady and Alvarez^23^ asked observers to recall the size of a single target circle that had been presented in a display that consisted of three different sets of circles, which could be grouped according to both size and colour. Interestingly, observers remembered the target circle as having been larger than it truly was when it was the same colour as the largest circles in the display, but as smaller when it shared the colour of the smallest circles^23^. Ensemble coding also influences memory for individual emotional expressions, as observers remember the expression shown by an individual face to be more alike the average expression of the group^46^. Faces less happy than the group average were remembered as being happier than they really were, while faces that were happier than the group average were remembered as being less happy^46^. Together, these findings suggest that visual working memory has a hierarchical structure, wherein the individual items from the display are encoded in reference to the summary statistics of the ensemble^45^, and when recalled, are incorrectly remembered as being more alike the ensemble average than they really were^23,46^.

Walker and Vul^16^ proposed that the cheerleader effect occurs as a consequence of the hierarchical structure of visual working memory, such that the recalled attractiveness of an individual face is biased toward the attractiveness of the ensemble representation that is summarised from the group^44^. Crucially, instead of being perceived to be of “average” attractiveness, faces with average characteristics are often perceived to be highly attractive^11,12,47,48^. In fact, digitally averaging individual faces together often results in a single average face that is perceived to be more attractive than each of the individual faces from which it was created^11,12,47^. Since digitally averaged faces appear to closely approximate the characteristics of the mentally summarised ensemble representation^37,38^, Walker and Vul^16^ suggested that ensemble representations might also be highly attractive, due to their average facial characteristics. In summary, Walker and Vul’s^16^ hierarchical encoding mechanism posits that the cheerleader effect occurs because the observer incorrectly remembers an individual face from a group as being similar to the ensemble representation^23,46^, which, due to its average facial characteristics, is more attractive than each individual face in the group^12^. However, even though the cheerleader effect is potentially consistent with the suggested hierarchical encoding mechanism, the role of such a mechanism in the effect has not been directly tested^13^. Therefore, the overarching aim of the current study was to investigate whether the cheerleader effect is caused by hierarchical encoding^16^.

## Experiment 1

Hierarchical encoding can only cause an individual to become more attractive in a group if the ensemble representation is more attractive than the individual being recalled. Walker and Vul^16^ hypothesised that the average facial characteristics of the ensemble representation might result in such high ratings of attractiveness. To explore what role, if any, hierarchical encoding has in the cheerleader effect, we investigated whether the cheerleader effect would occur when individual faces were presented in a group condition that, when summarised, should produce an ensemble representation that lacks the average facial characteristics that it requires to become highly attractive.

In a between-participants design, we randomly assigned observers to complete one of two cheerleader effect tasks. These two tasks differed in the degree of variance present between the three faces in each group (one target face, two distractor faces). In the *identical-distractors* task, observers rated the attractiveness of target faces that were presented once alone as a portrait image, and once in a group where the two distractor images were *identical* images of the target face. Crucially, there can be no variance between identical photographs of the same person. As such, the ensemble representation that is summarised from a group of identical faces should lack average facial characteristics and simply resemble the target face being recalled.

Conversely, observers in the *self-distractors* task rated the attractiveness of target faces that were presented once alone, and once in a group where the two distractor images were *different* photographs of the target identity. Unlike identical photographs, there can be substantial variance between different photographs of the same person due to random factors such as lighting, head orientation, and expression. This variability often results in observers classifying different photographs of the same person as being of two entirely different people^49^. As a result of this variability, different photographs of the same person can be digitally averaged to create a face with average characteristics^50^. This finding suggests that different photographs of the same person can also be mentally summarised to create an ensemble representation with average facial characteristics.

In both tasks (identical-distractors, self-distractors), observers also made attractiveness ratings for the same target faces when they were seen in a group with two unique distractor faces (the control condition)^13,16^. We predicted that the cheerleader effect would occur in each control condition, because the three unique faces can be summarised to create an ensemble representation with highly attractive, average facial characteristics^13,16^. For the same reason, we predicted that the cheerleader effect would also occur in the self-distractors condition, which featured three different images of the same person. Conversely, we did not expect the cheerleader effect to occur in the identical-distractors condition, because three identical photographs of the same person lack the variance needed to create an ensemble representation with average facial characteristics.

## Method

The cheerleader effect is presented as a change score, calculated by subtracting the attractiveness ratings given to the target faces when they are seen alone, from the attractiveness ratings they receive in a group^13,16,51^. A positive change value indicates that a face has become more attractive in a group, whereas a negative value represents a decrease in attractiveness. A one sample *t*-test is then used to determine whether the change in attractiveness differs from zero and is statistically significant^13,16^. Using this analytic approach, we have previously shown that the cheerleader effect occurs with a medium effect size (*d* = 0.60)^13^. An *a priori* power analysis indicated that a sample of 27 participants was required to achieve 85% power to detect an effect of *d* = 0.60 in a two-tailed, one sample *t*-test with an alpha of 0.05^52^.

All research reported in the current study was approved by, and conducted in accordance with the guidelines of, the Social and Behavioural Research Ethics Committee of Flinders University. All participants were recruited from Flinders University, gave their written informed consent prior to testing, were debriefed upon completion, and received course credit or $10 AUD for their participation. In total, each testing session lasted approximately 20–25 minutes. (Due to the relatively brief nature of these cheerleader effect tasks, participants in the self-distractors task in Experiment 1, and all participants in Experiment 2, completed a single additional task that was unrelated to the aims of the current research. In these circumstances, task order was counterbalanced between participants).

### Participants

Sixty-four participants (48 females, *M*_*age*_ = 23.58, *SD* = 9.73) were recruited to participate in either the identical-distractors or the self-distractors task. Prior to analysis^13^, exclusion criteria were adopted to exclude all data from participants with a cheerleader effect further than 3 *SD* from a condition mean (*n* = 2), as well as from those who failed to follow task instructions (*n* = 2). Additionally, participants with incomplete data due to technical failure were excluded from analysis (*n* = 1). The final sample in the identical-distractors task consisted of 28 participants (20 females, *M*_*age*_ = 26.68, *SD* = 13.76). The final sample in the self-distractors task consisted of 31 participants (23 females, *M*_*age*_ = 21.13, *SD* = 3.46).

### Apparatus

All experiments in the current study were programmed using E Prime 2.0 (Psychology Software Tools, Pittsburgh, PA), and presented on a 22” monitor (1680 × 1050) running at 60 Hz. Stimuli were viewed at an approximate distance of 500 mm. In each experiment, individual portraits were presented in the centre of the display (70 mm × 80 mm; 8.00° × 9.15°). Group images were created by presenting three individual portraits side by side (210 mm × 80 mm; 23.72° × 9.15°)^13,16^. The position of the target and distractor faces in the group (left, centre, right) was pseudo-randomly assigned to ensure that target faces appeared with equal frequency in each position within the group^13^. In experiments that required the same target face to be presented in multiple group conditions (Experiments 1 and 3), the target appeared in the same position within both groups.

### Stimuli

#### Identical-Distractors

The face stimuli used in the identical-distractors task came from the stimulus set created by Carragher, *et al*.^13^. This stimulus set was created by querying an online search engine with the search term “Bridesmaids”, in order to collect images of female faces in groups. Individual faces were then closely cropped from the original group image to create individual portraits. In total, this stimulus set contained 320 unique female faces showing happy expressions (as determined by the researchers). Most faces appeared to be of Caucasian ethnicity and between 20 and 40 years of age.

**Figure 1:**
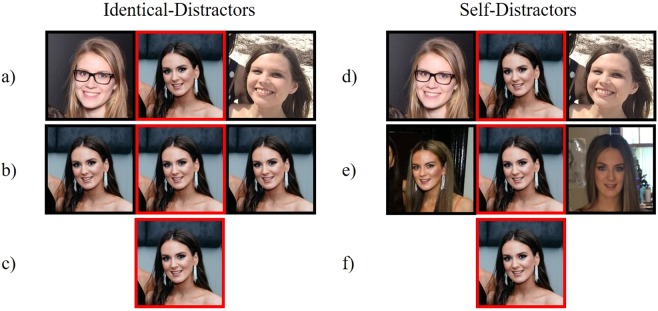
Stimulus configurations for the identical-distractors and self-distractors tasks in Experiment 1. Identical-Distractors Task (**a**) control group (**b**) identical-distractor group (**c**) alone trial. Self-Distractors Task (**d**) control group (**e**) self-distractor group (**f**) alone. The target face was identified by a red frame (centre). [Due to copyright restrictions, the faces in the figures throughout the manuscript are representative of those in the stimulus set but were not themselves shown in the experiment].

From this stimulus set, 105 faces were randomly selected to be presented as target faces in the identical-distractors task. The remaining faces in the stimulus set were randomly presented as distractor faces in the control condition (see Fig. 1a). Identical-distractor groups were created by simultaneously presenting the target image three times, side by side (see Fig. 1b). The identical-distractors task consisted of 105 control, 105 identical-distractor, and 105 alone trials, which were intermixed and randomised.

#### Self-Distractors

A new stimulus set was created specifically for the self-distractors task, as multiple images of the same identity were required. We collected 3 different images of 225 unique identities from online sources, by sampling photographs of female athletes. Female athletes were selected for the stimuli set because many labelled images of athletes are available online, but they were unlikely to be familiar to participants (indeed, no participant spontaneously claimed to have recognised an identity from the stimulus set)^53^. These faces were also all female and showed happy expressions. Most faces appeared to be of Caucasian ethnicity and between 20 and 40 years of age. All images were cropped to remove any external cues related to the individual’s profession or identity (e.g., sporting equipment, corporate logos).

From this stimulus set, 75 individuals were randomly selected as target identities. From the three images available for each target identity, one was randomly selected to be the target face, while the two remaining images were presented as the distractor faces in the self-distractors condition (see Fig. 1e). The distractor faces in the control condition were sampled by randomly selecting a single image from each of the 150 remaining identities in the stimulus set. In total, the self-distractors task consisted of 75 control, 75 self-distractor, and 75 alone trials, which were intermixed and randomised. Notably, the self-distractors task had fewer trials than the identical-distractors task simply because there were fewer faces in the stimulus set; however, since the analyses were conducted using the average attractiveness ratings made by participants, the reduced number of trials was unlikely to adversely affect the reliability of the results.

### Procedure

**Figure 2:**
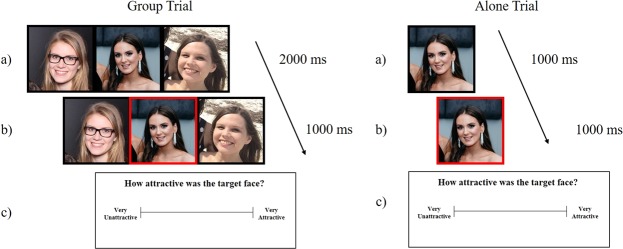
An example of the trial procedure for a control group trial, and an alone target trial. (**a**) The image is initially presented without the target cued (**b**) The target is cued from the image by the red frame [centre] (**c**) an attractiveness rating is made along the visual analogue scale.

The trial procedure was the same for both cheerleader effect tasks, and unless otherwise noted, for all experiments reported in the current study. Participants were asked to rate the attractiveness of the target face in each group, which was identified after a brief interval by a red frame (see Fig. 2). Participants completed six practice trials to familiarise themselves with the task. Initially, group images were presented without the target identified for 2000 ms, and each face was surrounded by a black frame. During this time, participants were encouraged to look at each face in the group. A red frame then appeared around the target face for an additional 1000 ms, after which time all images disappeared from the screen. During alone trials, the target face was presented for 1000 ms with a black frame, and then for 1000 ms with a red frame, before disappearing from the screen. Participants gave their attractiveness rating for the target face via mouse click along a visual analogue scale, which ranged from “Very Unattractive” (0%) to “Very Attractive” (100%; width = 192 mm; 21.74°)^13^. This procedure was first introduced by Walker and Vul^16^ (Experiment 4) and was used by Carragher, *et al*.^13^.

## Results

**Figure 3:**
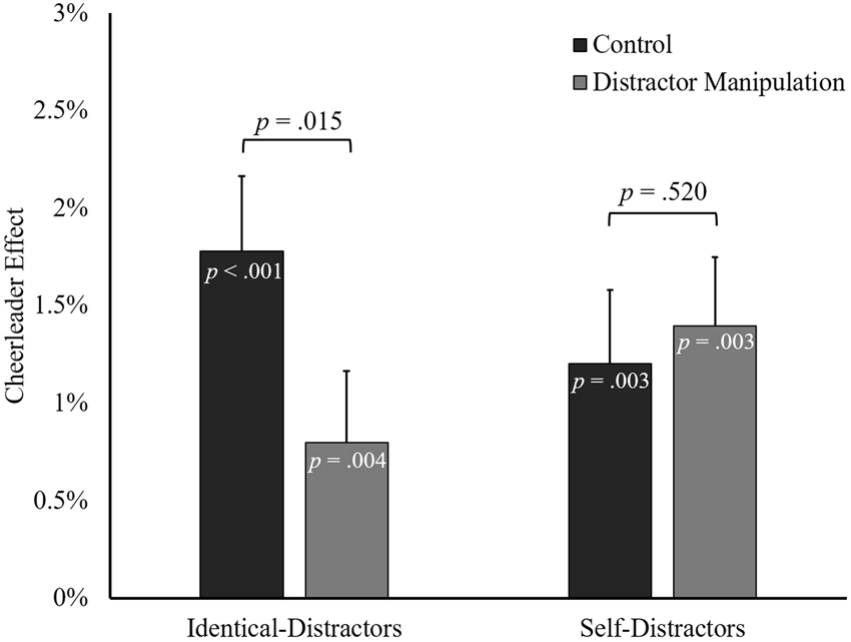
The cheerleader effect in the control and distractor manipulation conditions, shown separately for participants in the identical-distractors and self-distractors tasks. Error bars represent the standard error of the mean.

Separate cheerleader effect measures were calculated for the two group conditions in each cheerleader effect task. A 2 × 2 repeated-measures ANOVA with group condition (control, distractor manipulation) as a within-participants factor, and cheerleader effect task (identical-distractors, self-distractors) as a between-participants factor, was used to investigate whether the variance between the faces in the group influenced the size of the cheerleader effect. The main effect of group condition was non-significant, *F*(1, 57) = 2.80, *p* = 0.100, ${\eta }_{p}^{2}$ = 0.047, as was the main effect of cheerleader effect task, *F*(1, 57) < 0.01, *p* = 0.983, ${\eta }_{p}^{2}$ < 0.001. Crucially, the interaction between group condition and cheerleader effect task was significant, *F*(1, 57) = 6.13, *p* = 0.016, ${\eta }_{p}^{2}$ = 0.097, (see Fig. 3). Separate analyses were conducted for each cheerleader effect task to investigate the nature of the significant interaction.

### Identical-Distractors Task

One sample *t*-tests were used to investigate whether the change to the perceived attractiveness of the target faces when presented in a group was statistically significant. Consistent with the cheerleader effect, faces were perceived to be significantly more attractive when presented in the control, *t*(27) = 4.71, *95%CI*[1.01, 2.56], *p* < 0.001, *d* = 0.89, and identical-distractors conditions, *t*(27) = 3.18, *95%CI*[0.28, 1.32], *p* = 0.004, *d* = 0.60. A paired-samples *t*-test was used to compare the size of the cheerleader effect between the two group conditions. The cheerleader effect was significantly larger in the control condition than in the identical-distractors condition, *t*(27) = 2.59, *95%CI*[0.20, 1.76], *p* = 0.015, *d* = 0.49.

### Self-Distractors Task

One sample *t*-tests showed that target faces were perceived to be significantly more attractive when presented in both the control, *t*(30) = 3.24, *95%CI*[0.45, 1.96], *p* = 0.003, *d* = 0.58, and self-distractors conditions, *t*(30) = 3.21, *95%CI*[0.51, 2.28], *p* = 0.003, *d* = 0.58. A paired-samples *t*-test indicated that the difference between the control and self-distractors conditions was non-significant, *t*(30) = 0.65, *95%CI*[−0.41, 0.79], *p* = 0.520, *d* = 0.12.

## Discussion

Target faces were perceived to be significantly more attractive in the control condition for both participant groups, replicating the cheerleader effect^13,16^. As predicted, the cheerleader effect also occurred in the self-distractors condition. Interestingly, the size of the effect did not differ significantly between the control and the self-distractors conditions, suggesting that the faces in the self-distractors groups contained a similar amount of variability as the faces of different identities in the control condition^49,50,54^. Notably, the cheerleader effect was significantly reduced in the identical-distractors condition, which is partially consistent with the predictions we derived from the hierarchical encoding mechanism^16^. The reduction to the cheerleader effect in the identical-distractors condition cannot be attributed to the three faces in the group sharing the same identity, because that was also true of the self-distractors condition. Instead, our findings are consistent with the hypothesis that the cheerleader effect was significantly reduced in the identical-distractors condition because these groups could not be summarised to create ensemble representations with average facial characteristics that were significantly more attractive than the target faces in the group.

Despite offering some support for the role of hierarchical encoding in the cheerleader effect, a small, but statistically significant, increase in attractiveness still occurred in the identical-distractors condition. Because there is no physiological variance between the images in the identical-distractors condition, the ensemble representation of such a group should lack the average facial characteristics that it requires to become highly attractive. Therefore, this finding raises the possibility that hierarchical encoding might not be necessary for the cheerleader effect to occur. Yet, a limitation of this experiment is that our interpretation of these results relies upon assumptions about the likely properties of the unseen, mentally summarised ensemble representation. We sought to address this limitation in Experiment 2, by explicitly testing whether the size of the cheerleader effect that each target face experiences is related to the attractiveness of the average face of the group that it appears within.

## Experiment 2

In Experiment 1, we found mixed support for the hierarchical encoding account of the cheerleader effect. To investigate whether the cheerleader effect is related to the characteristics of the ensemble representation, a new sample of observers completed the control cheerleader effect task. During this task, observers also gave attractiveness ratings for faces that were created by digitally averaging the three faces in each group together. To avoid confusion from similar terminology, the digitally averaged face stimuli in the current experiment are henceforth referred to as “morph faces”. These morph faces were used to approximate the characteristics of the ensemble representation for each group image^37–39^. The sensitivity with which observers mistakenly recognise the digitally averaged face of a seen group in tasks measuring the ensemble coding of identity^37,38^, strongly suggests that digitally averaging the individual faces in the group together produces a morph face that closely resembles the ensemble representation that is mentally summarised by the observer.

The attractiveness ratings given to the morph faces in the current experiment were used to test two explicit predictions derived from the hierarchical encoding mechanism^16^. First, we investigated whether the morph face of each group was perceived to be more attractive than each of the three individual faces used to create it^12,47^. Second, we tested whether the size of the cheerleader effect that each target face experienced was related to the attractiveness rating given to the morph face of the group it appeared within. Consistent with an effect of hierarchical encoding, we predicted that the individual faces that were much less attractive than the morph face would experience the largest increase in attractiveness, whereas any individual face that was more attractive than the morph face would become less attractive in a group^46^.

## Method

### Participants

Thirty-one participants (25 females, *M*_*age*_ = 19.97, *SD* = 4.12) were recruited to participate in Experiment 2. All participants completed the task as instructed, and no cheerleader effect data fell further than 3 *SD* from the condition mean. As such, no data were excluded from analysis.

### Stimuli

The naturalistic face stimuli from Experiment 1^13^ were too variable in their orientation and expression to create morph faces that appeared seamlessly realistic. Consequently, we selected 30 female faces with neutral expressions from the Karolinska Directed Emotional Faces database (KDEF)^55^ to use in the current experiment (all faces from the AF-NES KDEF image set were used except AF04NES, AF10NES, AF18NES, AF23NES, and AF31NES). These 30 faces were randomly sorted to create 10 group images with 3 faces in each. Unlike Experiment 1, we collected a cheerleader effect measure for each face in the group, to maximise the number of possible trials with 10 group images. As such, each group image was presented three times during the experiment, and a different face was identified as the target each time^16^. With this single exception, the trial procedure for the current experiment was identical to that of Experiment 1.

**Figure 4:**
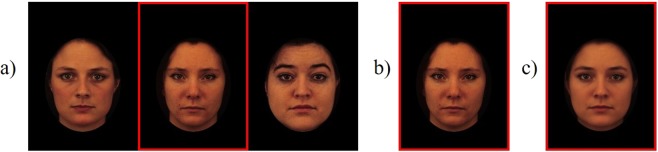
Example stimulus configurations in Experiment 2 (**a**) group trial (**b**) alone trial (**c**) morph face trial. The figure shows images AF01NES, AF02NES and AF03NES from the KDEF^55^, as well as the morph face created from these three images (**c**).

The morph face for each group image was created using FantaMorph Deluxe 5.4.8 (Abrosoft, 2016). The three faces in each group were weighted equally in the averaging procedure to create a morph face that accurately depicted the average facial characteristics of each group (see Fig. 4c). A black mask was placed around all face stimuli to occlude the external features of the face (e.g., hair, neck), where evidence of digital averaging could potentially be noticed in the morph faces (see Fig. 4). In total, the experiment consisted of 30 group, 30 alone, and 10 morph face trials that were intermixed and randomised.

### Analysis

To investigate whether hierarchical encoding causes the cheerleader effect, we created a second change measure, called the “*ensemble effect*”. This ensemble effect measure was calculated for each target face by subtracting the attractiveness rating given to the face when it was seen alone (Fig. 4b), from the attractiveness rating given to the morph face of the group that it was presented in (Fig. 4c). Therefore, ensemble effect shows how much more attractive the morph face was, compared to each target face that it was made from. We hypothesised that hierarchical encoding would be demonstrated by a *positive* correlation between ensemble effect and the size of the cheerleader effect. A positive correlation would show that the target faces that were much less attractive than the morph face (i.e., those with positive ensemble effect scores) received the largest increases in attractiveness, whereas any target face more attractive than the morph face (i.e., those with a negative ensemble effect) would be percieved to be less attractive in a group.

## Results

### Morph Faces

**Figure 5:**
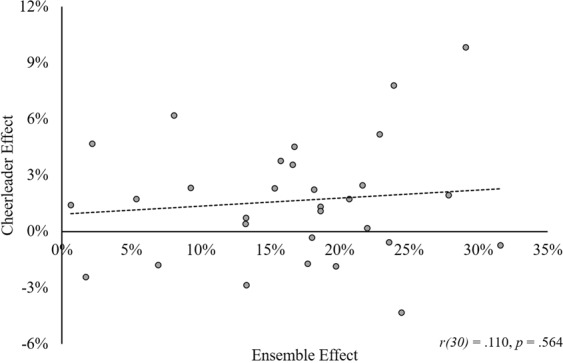
The relationship between the size of the cheerleader effect and the ensemble effect measure for each target face in the stimulus set. Positive ensemble effect scores indicate that the morph face was more attractive than the individual target face.

First, we examined whether the morph faces were perceived to be significantly more attractive than the individual faces from which they were made. A paired samples *t*-test showed that participants perceived the morph faces to be significantly more attractive (*M* = 52.53, *SD* = 13.54) than the original target faces (*M* = 35.96, *SD* = 11.49), *t*(30) = 9.96, 95%*CI*[13.17, 19.97], *p* < 0.001, *d* = 1.79. Further examination of these attractiveness ratings showed that all 10 morph faces were rated to be more attractive than the 3 original faces from which each was made (M_*increase*_ = 16.57%, SD = 8.04; Range = 0.65%, 31.59%; see Fig. 5). These findings support the hypothesis that the ensemble representation is perceived to be more attractive than each individual face in the group.

### Cheerleader Effect

A one sample *t*-test showed that the target faces were perceived to be significantly more attractive in a group compared to alone (*M* = 1.64%, *SD* = 2.30), *t*(30) = 3.97, 95%*CI*[0.79, 2.48], *p* < 0.001, *d* = 0.71, replicating the cheerleader effect.

### Hierarchical Encoding

We tested whether hierarchical encoding contributes to the cheerleader effect by investigating the relationship between the size of the cheerleader effect and the ensemble effect measure for each target face. In contrast to our predictions of a significant positive correlation between the two measures, the relationship between ensemble effect and the cheerleader effect was non-significant, *r*(30) = 0.110, *95%CI*[−0.261, 0.452], *p* = 0.564. This result suggests that the size of the cheerleader effect that each target face experiences is not related to the attractiveness of the morph face of the group (see Fig. 5).

## Discussion

Each morph face was perceived to be significantly more attractive than the three individual faces from which it was created, replicating the findings of Langlois and Roggman^12^. Furthermore, the average increase in attractiveness for an individual face when seen in a group was approximately 1.5–2.0%, replicating the size of the cheerleader effect in the two control conditions in Experiment 1. Yet, our data also clearly show that the size of the cheerleader effect that each target face experienced was not related to the attractiveness of the morph face of the group. Those target faces that were much less attractive than the morph face of the group did not receive the largest increase in attractiveness, which is inconsistent with hierarchical encoding. Therefore, despite showing that the morph face of each group was more attractive than each target face, and that the target faces were remembered to be more attractive in a group than they were alone, our results suggest that these two findings are unrelated.

Taken together, Experiments 1 and 2 do not provide strong support for the proposition that the cheerleader effect is caused by hierarchical encoding^16^. However, we cannot rule out the possibility that these experiments have failed to adequately capture the underlying hierarchical encoding process. For example, in Experiment 1 we hypothesised that because the group images in the identical-distractors condition did not contain any variance, they could not be summarised to produce an ensemble representation with average facial characteristics. Yet, even though there is no physical variance present between identical images of the same face, it is possible that internal noise from the observer is introduced during the encoding of the individual stimuli. If this internal noise is uncorrelated across the identical faces in the group, the ensemble representation might still acquire the some average facial characteristics that could cause the cheerleader effect^21^. Additionally, it is also possible that the ensemble effect measure created in Experiment 2 was not sensitive enough to detect evidence of hierarchical encoding in the cheerleader effect. To address these alternative explanations for the absence of evidence for hierarchical encoding in Experiments 1 and 2, we conducted one final experiment to investigate whether the cheerleader effect would occur for faces that were presented in groups with non-human distractors.

## Experiment 3

The primary aim of Experiment 3 was to test whether the cheerleader effect occurs for a face shown in a group with non-human distractor images. To this end, images of *houses* were presented in place of the distractor faces in the group (house-distractors condition). Houses were selected to replace the distractor faces because they are an easily identifiable, non-human stimulus that belong to a homogenous item category^3^. A significant increase in attractiveness for a face presented amongst a group of houses would provide the strongest evidence to date that hierarchical encoding does not cause the cheerleader effect, because the ensemble representation of such a group, to the extent that a coherent summary representation can be created from an ensemble of heterogenous items, would not be a human face. Without an ensemble representation that is a human face, any increase in attractiveness for a face seen in a group with house distractors cannot be attributed to hierarchical encoding.

In this experiment, we also investigated whether the cheerleader effect itself is specific to human faces. Throughout the experiment, participants were also asked to make attractiveness judgments about target *houses*, which were presented once alone, and once in a group with two distractor *houses* (i.e., a control house group). Curiously, previous investigations have shown that fruit^56^ and other food products^57^ are perceived to be more attractive when they are seen within a group of similar items compared to alone. Therefore, we predicted that the cheerleader effect would also occur for images of houses. A significant cheerleader effect for target houses would provide the first evidence that the cheerleader effect generalises beyond human faces, and therefore does not rely on a face specific processing mechanism.

## Method

### Participants

Twenty-nine participants (25 females, *M*_*age*_ = 22.52, *SD* = 9.28) were recruited for Experiment 3. As all data were within 3 *SD* of the condition mean and all participants completed the task as instructed, no data were excluded from analysis.

### Stimuli

**Figure 6:**
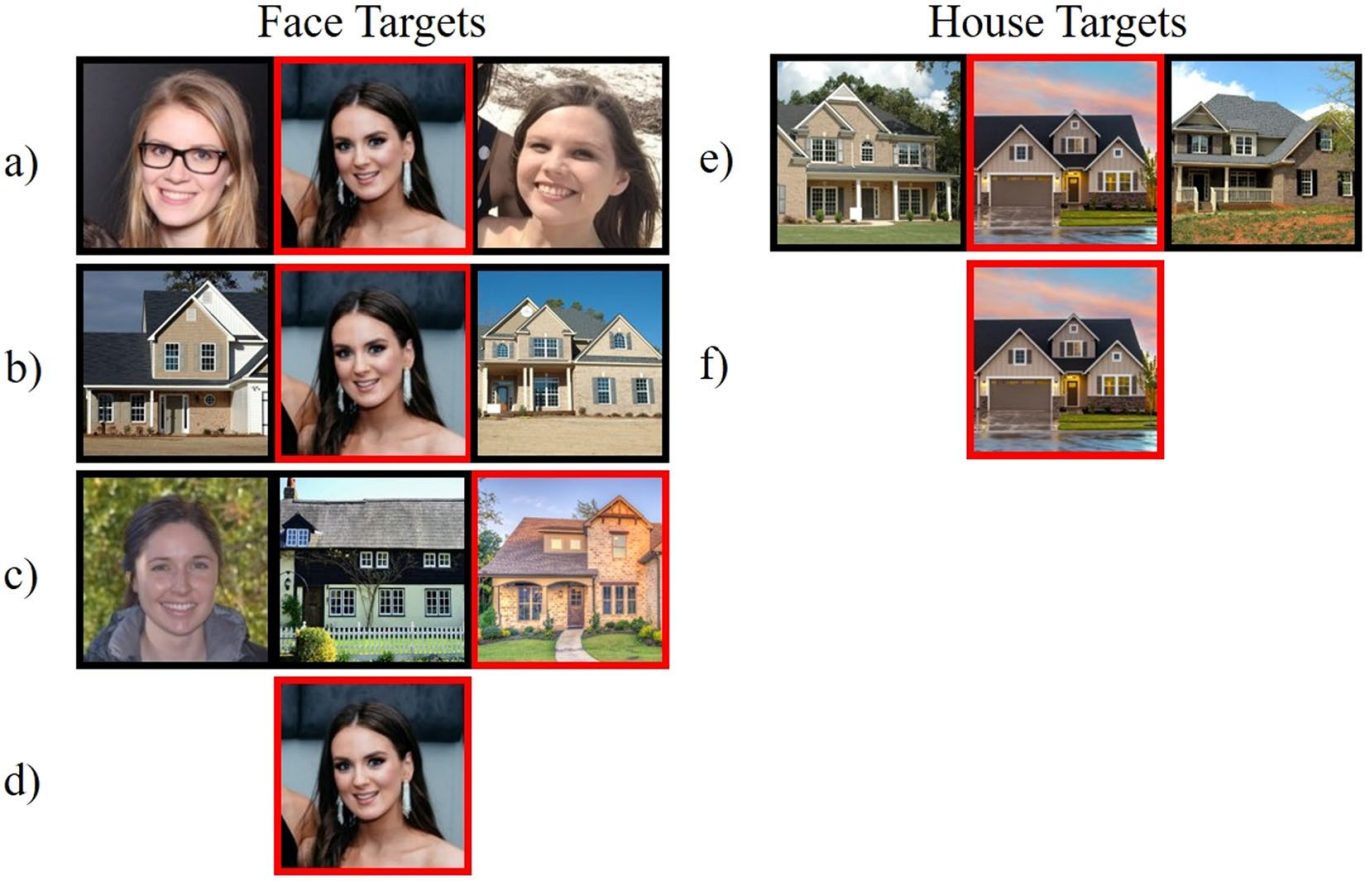
Stimulus configurations in Experiment 3. Face Targets (**a**) control trial (**b**) house-distractors trial (**c**) filler trial [target right] (**d**) alone face trial. House Targets (**e**) house-target trial (**f**) alone house trial. [Due to copyright restrictions, the house images in this figure are representative of those in the stimulus set but were not themselves shown in the experiment. The house images in this figure were downloaded from www.pexels.com and are published in accordance with the Pexels License].

A new stimulus set consisting of images of houses was collected from various real estate websites. All houses included in the stimulus set were two-story, featured windows on the top level of the house, and were free of other notable stimuli (e.g., people, cars). From this stimulus set, 60 target houses were randomly selected to be presented twice: once in a group with two unique distractor houses (house-target condition), and once alone (alone house trial; see Fig. 6). The procedure for responding to house targets was identical to that for faces.

From the stimulus set of Carragher, *et al*.^13^, 60 target faces were randomly selected to be presented 3 times: once in a control group, once in a group where the distractor images were houses (house-distractors condition), and once alone. To discourage participants from fixating the only human face in a house-distractors group, 60 additional filler trials were included in the task, wherein a house was identified as the target in a group that contained a single human face and two houses (see Fig. 6c). These filler trials were not of theoretical importance and were discarded prior to analysis. Both target *face* and target *house* trials were intermixed and randomised throughout the experiment, which consisted of 60 control, 60 house-distractors, 60 filler, 60 alone face, 60 house-target and 60 alone house trials. The trial procedure was identical to Experiments 1 and 2.

## Results

### Cheerleader Effect

One sample *t*-tests showed that faces were significantly more attractive when presented in groups in both the control (*M* = 1.48%, *SD* = 2.43), *t*(28) = 3.27, *95%CI*[0.55, 2.40], *p* = 0.003, *d* = 0.61, and house-distractors conditions (*M* = 0.75%, *SD* = 1.67), *t*(28) = 2.42, *95%CI*[0.12, 1.39], *p* = 0.022, *d* = 0.45. A paired-samples *t*-test showed that the cheerleader effect was significantly larger in the control condition than the house-distractors condition, *t*(28) = 2.14, *95%CI*[0.03, 1.42], *p* = 0.041, *d* = 0.40.

### House Targets

A one sample *t*-test showed that target houses were significantly more attractive when shown in a group of houses compared to alone (*M* = 1.17%, *SD* = 2.25), *t*(28) = 2.81, *95%CI*[0.32, 2.02], *p* = 0.009, *d* = 0.52.

## Discussion

The cheerleader effect occurred in the control condition, replicating the results of Experiments 1 and 2. Interestingly, although significantly reduced, the cheerleader effect remained statistically significant when target faces were shown in the house-distractors condition. The results of the present study are conceptually similar to those of Experiment 1, wherein the cheerleader effect was significantly reduced, but not eliminated, when target faces were presented in the identical-distractors condition. Notably, neither the house-distractors nor identical-distractors groups could be summarised to create an ensemble representation with average facial characteristics, further suggesting that the cheerleader effect is unrelated to the summary representation of the group.

Interestingly, target houses were also perceived to be significantly more attractive when shown in a group of houses compared to when they were seen alone. This finding is consistent with previous studies that have found fruit^56^ and other food products^57^ are perceived to be more attractive in a group compared to alone. A significant increase in attractiveness for a non-human object in a group suggests that the cheerleader effect is not caused by a face specific processing mechanism.

## General Discussion

In the first empirical account of the cheerleader effect, Walker and Vul^16^ proposed that the effect was caused by the hierarchical structure of visual working memory^23^. Consistent with the predictions derived from this hierarchical encoding mechanism, we found the cheerleader effect to be significantly larger in group conditions that could be summarised to create an ensemble representation with average facial characteristics (e.g., control, self-distractors), compared to those group conditions that could not (e.g., identical-distractors, house-distractors). In this respect, our results could be interpreted as providing partial support for the role of hierarchical encoding in the cheerleader effect^16^. However, several of our findings are inconsistent with this mechanism all together. Experiment 2 showed that the change in attractiveness for an individual face seen in a group was not related to the attractiveness rating given to the morph face of the group, which was used to approximate the characteristics of the ensemble representation. Furthermore, the cheerleader effect was only reduced, but not eliminated, in group conditions where the summary representation of the group should not have average facial characteristics (e.g., identical-distractors), or even have been a human face (e.g., house-distractors). Therefore, the small increase in attractiveness for faces in these group conditions strongly suggests that hierarchical encoding is not necessary to observe the cheerleader effect.

Interestingly, Walker and Vul^16^ also noted that two findings in their original study were potentially inconsistent with a hierarchical encoding mechanism. First, increasing the number of distractor faces in the group did not cause a larger cheerleader effect, even though the characteristics of the ensemble representation should become increasingly average, and more attractive, as additional faces are added to the group image^16^. Second, blurring the face stimuli did not result in a larger cheerleader effect, even though the introduced uncertainty should disproportionately affect the encoding of the individual faces, compared to the ensemble representation^16^. Similarly, Luo and Zhou^44^ also identified limitations to the hierarchical encoding account of the cheerleader effect, while investigating ensemble coding in judgments of facial attractiveness. Notably, that observers showed a tendency to extract ensemble representations that were slightly *less* attractive than average attractiveness of each group^44^. Specifically referring to the cheerleader effect, Luo and Zhou^44^ reasoned that if the ensemble representation was perceived to be highly attractive, observers should systematically overestimate, not underestimate, the average attractiveness of the group. Therefore, although the current study is the first to directly investigate the underlying cause of the cheerleader effect, the limitations of the hierarchical encoding mechanism have been alluded to previously^16,44^. Our findings provide the strongest evidence to date that hierarchical encoding appears to only make a minimal contribution to the cheerleader effect, if any at all. In light of these findings, we believe that future research should be directed toward exploring other possible causes of the cheerleader effect.

To identify the underlying cause of the cheerleader effect, one might begin by considering other instances in which social context influences judgments of facial attractiveness. For example, the “group attractiveness effect”^15^ occurs when observers overestimate the attractiveness of a whole group of faces (e.g., “how attractive is this group?”), compared to the mathematical average of the attractiveness ratings given to each face in the group. Using eye-tracking, van Osch, *et al*.^15^ showed that observers spent more time gazing at the most attractive faces in each group, suggesting that selectively attending to only the most attractive faces caused observers to overestimate the attractiveness of the whole group. As noted by Carragher, *et al*.^13,51^, it is possible that the cheerleader effect might also occur due to selective attention; rather than being biased toward the attractiveness of the ensemble representation, observers might instead give attractiveness ratings to the target face that are influenced by the most attractive face in the group. However, while a selective attention mechanism could potentially explain the conventional cheerleader effect^13,16^, this mechanism cannot account for the increase in attractiveness found for target faces in the identical-distractors condition (since the faces were necessarily of equal attractiveness). Therefore, it appears unlikely that selective attention toward the most attractive faces causes the cheerleader effect.

Similarities can also be seen between the cheerleader effect and “mate choice copying”, an evolutionary phenomenon that occurs when an observer increases their preference for a potential mate, after learning that the potential mate is desired by others^14,58^. Demonstrated by male and female observers^59,60^, opposite-sex faces (the potential mates) are perceived to be more attractive when seen with an attractive partner (the same-sex as the observer), compared to an unattractive partner^59,61^, or alone^58^. Consistent with the theory that mate choice copying is used to infer unobservable qualities in a potential mate^14,58^, female observers attribute more desirable personality traits, including being more trustworthy and caring, to the same male face when seen with a female partner compared to alone^62^. Perhaps the cheerleader effect also occurs through a process of social inference, wherein observers use the presence of other faces in the group to infer that the evaluated individual likely possesses desirable characteristics, such as being friendly or kind, which consequently increases their perceived favourability to the observer^62^. Importantly, this type of social learning is not specific to instances of mate selection^63^, or even to the presence of human faces, since favourable characteristics, including attractiveness, are attributed to faces shown with possessions that signal wealth or social status^64–70^. Therefore, a social inference mechanism could also explain why a small increase in attractiveness is found for faces shown among groups of houses. However, such a mechanism does not clearly explain the increase in attractiveness found for a house seen among other houses. Although the simplest explanation for the cheerleader effect is one that relies on a single mechanism, at present, the effect does not appear to be consistent with a single mechanism of hierarchical encoding, selective attention, or social inference. As such, we should consider the possibility that multiple, separate mechanisms contribute to the cheerleader effect.

Although our findings are inconsistent with a cheerleader effect that is caused by a single hierarchical encoding mechanism, it should be considered that ensemble coding, the prerequisite process for hierarchical encoding, occurs rapidly, if not automatically, upon viewing a scene^25,27,33,34^. Therefore, one possibility that cannot be discounted, is that two sperate mechanisms, of which one is hierarchical encoding, contribute to the increase in attractiveness that is typical of the cheerleader effect. In support of this proposition, our findings show that the size of the cheerleader effect fluctuates depending on whether it is possible for hierarchical encoding to occur. An increase in attractiveness of approximately 1.5% is found in group conditions that can be summarised to create an ensemble representation with average facial characteristics, whereas a significantly smaller, but still statistically significant, increase in attractiveness of approximately 0.8% is observed in group conditions that should not be subject to hierarchical encoding. If the cheerleader effect is caused by two separate mechanisms, these results suggest that approximately half of the increase in attractiveness that is typical of the cheerleader effect can be attributed to hierarchical encoding. Notably, since an increase in attractiveness was found for faces in groups of non-human stimuli, this hypothetical second mechanism would not necessarily be specific to the processing of faces. One possibility that should be considered in future research is that a social inference mechanism causes an initial increase in attractiveness for a face that is seen in a group, which can then be further increased if it is possible for hierarchical encoding to occur.

Interestingly, the possibility that the cheerleader effect is caused by two concurrent mechanisms was also suggested very recently by Ying, *et al*.^19^. Using a psychophysical paradigm, in which observers made a two-alternative forced-choice discrimination about the attractiveness of the target face (attractive or unattractive), Ying, *et al*.^19^ found that a cheerleader effect occurred regardless of the attractiveness of the distractor faces in the group. However, the largest increase in attractiveness occurred for faces in unattractive groups, and the smallest increase for faces in attractive groups^19^. To explain their results, Ying, *et al*.^19^ suggested that the cheerleader effect was the result of two separate mechanisms; “social positive effect”, which causes an initial increase in attractiveness, and an effect of contrast, which modulates the size of the attractiveness increase. Though not clearly defined, social positive effect appears to be comparable to the social inference mechanism described in the current study, in which observers use social cues when evaluating the target face. Unlike social positive effect, the contrast effect observed by Ying, *et al*.^19^ does not always increase the attractiveness of an individual. Rather, the size of the cheerleader effect appears to rely on the interaction between the attractiveness of the target face and that of the group. Although the current study cannot examine the role a contrast mechanism (since target and distractor faces were always randomly selected from the stimulus set), we believe that future research would be well directed to further investigate these two-mechanism accounts of the cheerleader effect.

This future research should also investigate the single discrepancy between the results of the current study and those of Ying, *et al*.^19^. Despite employing different methodologies, both the present study and Ying, *et al*.^19^, found that the cheerleader effect was significantly attenuated in an identical-distractors condition relative to the control condition. However, the small increase in attractiveness for the identical-distractors condition reached statistical significance in the current study, which was not found by Ying, *et al*.^19^. In the two-mechanism framework of the cheerleader effect proposed above, understanding whether an increase in attractiveness occurs for faces among identical-distractors will clarify the nature of the social inference mechanism. From the null-result in their identical-distractors condition, Ying, *et al*.^19^ concluded that variance between the faces in the group *is* necessary to activate the initial social positive effect mechanism. Conversely, our results suggest that variance between the faces *is* *not* necessary to observe a small increase in attractiveness. Although it is possible that social inference contributes to the cheerleader effect, our results leave open the possibility that this effect is simply one of mere presence, wherein an increase in attractiveness is found by virtue of appearing in a group made from any kind of images. This interpretation would be consistent with the significant cheerleader effect we observed for faces shown among houses, and for houses in groups of other houses. Future research is needed to understand whether the mere presence of other images is all that is required for the cheerleader effect to occur.

The uncertainty surrounding the cause of the cheerleader effect should not be misconstrued to suggest that the effect is unreliable. Across three experiments, the cheerleader effect was shown to be a robust phenomenon that consistently resulted in the same individual being rated to be approximately 1.5–2.0% more attractive when seen in a group compared to when seen alone. Our results strongly suggest that the cheerleader effect is not consistent with a single hierarchical encoding mechanism^16^, and that future research is necessary to re-examine the underlying cause of the effect. Furthermore, our results suggest that the cheerleader effect is not caused by a face specific processing mechanism, since an increase in attractiveness was also shown to occur for non-human items shown in non-human groups. A mechanism that might warrant further investigation is social inference^19,62^, wherein the cheerleader effect is the result of the positive characteristics that are attributed to an individual when they appear to be endorsed by others, or are associated with items that signal social status. Additional research might also investigate the possibility that the cheerleader effect is the result of two separate mechanisms that work concurrently to influence the attractiveness of an individual in a group, a suggestion made both in the current study and recently by Ying, *et al*.^19^. The robust nature of the cheerleader effect suggests that social context is an important factor to consider in the ongoing study of facial attractiveness, and raises the possibility that other trait impressions might also be influenced by social context.

## Data Availability

All data reported in the current study are available in the Open Science Framework repository [https://osf.io/je5u7/].
